# Rapid awake airway stabilization by direct tracheal intubation through an open cervical wound in severe laryngotracheal injury: a case report

**DOI:** 10.1186/s12893-026-03764-9

**Published:** 2026-04-20

**Authors:** Yajie Zhao, Hansheng Liang, Yi Feng

**Affiliations:** Department of Anesthesiology, Peking University People’s Hospital, No.11 Xizhimen South Street, Xicheng District, Beijing, 100044 P. R. China

**Keywords:** Airway management, Laryngotracheal trauma, Penetrating neck injury, Wound intubation, Case report

## Abstract

**Background:**

Penetrating neck injuries with open laryngotracheal disruption are rare but represent an extreme airway emergency in which standard airway management algorithms may be unsafe due to profound anatomical distortion. In such situations, airway control must be tailored to the extent of structural disruption.

**Case presentation:**

We report a 38-year-old woman who sustained a severe anterior cervical stab wound resulting in complete laryngotracheal disruption, including transection of the thyroid cartilage and rupture of the cricoid cartilage. Conventional oral intubation and rapid sequence intubation were contraindicated due to distorted anatomy, and immediate tracheotomy was not feasible under awake conditions. Therefore, awake tracheal intubation through the open cervical wound was deliberately selected as a nonconventional airway management strategy to achieve rapid airway stabilization. Following successful airway control, the patient underwent definitive tracheostomy and surgical reconstruction, with successful decannulation four weeks later. Follow-up examinations at three and six months demonstrated normal vocal cord mobility and showed no evidence of tracheal stenosis on flexible laryngoscopy.

**Conclusions:**

This case highlights the importance of anatomy-driven and innovative airway decision-making and suggests that direct wound intubation may expand the available airway management options in carefully selected patients with severe penetrating laryngotracheal trauma.

**Supplementary Information:**

The online version contains supplementary material available at 10.1186/s12893-026-03764-9.

## Background

Penetrating neck injuries (PNIs) are uncommon but potentially life-threatening, with reported mortality rates ranging from 5% to 20%, largely attributable to airway compromise, major vascular injury, and concomitant aerodigestive tract damage [1, 2]. Laryngotracheal disruption represents a particularly severe subset of PNIs and poses unique clinical challenges, as acute hemorrhage, extensive subcutaneous emphysema, and profound anatomical distortion may render conventional airway management techniques unsafe or ineffective. Early recognition of airway instability and timely airway management are therefore paramount, as failure to promptly secure a definitive airway can rapidly lead to catastrophic hypoxia and fatal outcomes.

In this clinical setting, airway management strategies must be individualized and guided by the degree of anatomical disruption, the presence of active bleeding, and the patient’s physiological status. Here, we report a case of severe penetrating neck injury with complete transection of the thyroid cartilage and rupture of the cricoid cartilage, in which a deliberate decision was made to perform awake tracheal intubation through the open cervical wound as a nonconventional airway management approach.

## Case presentation

**Fig. 1:**
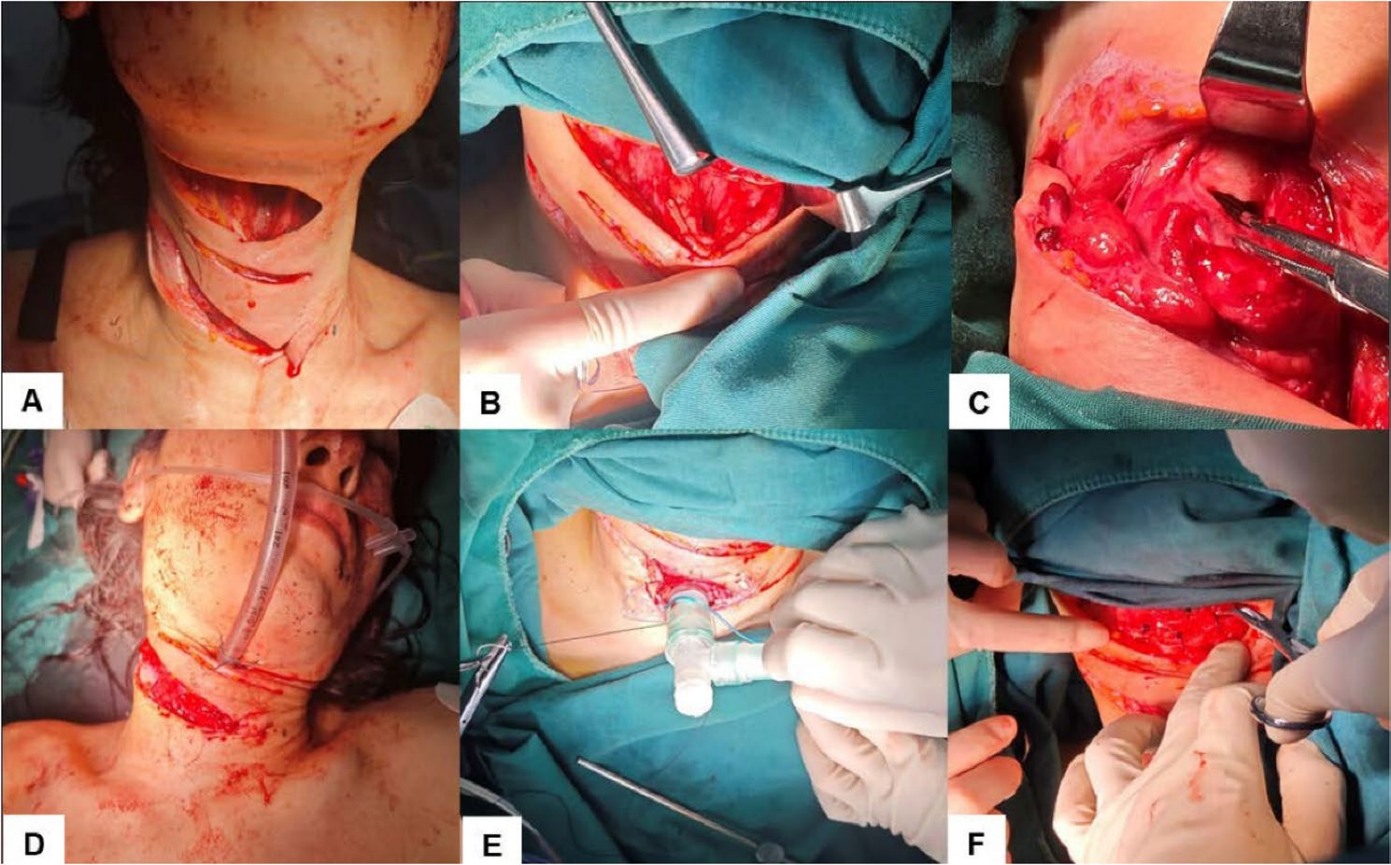
Emergency airway management for laryngotracheal disruption. **A** Physical examination showed three skin wound on the anterior neck. **B** Complete thyroid cartilage transection. **C** Cricoid cartilage rupture. **D** Tracheal intubation through the open cervical wound for emergency airway management. **E** Tracheostomy performed at the level of the second to third tracheal rings. **F** Thyroid cartilage reconstruction

A 38-year-old woman with no relevant past medical history was admitted to the emergency department after sustaining a penetrating neck injury caused by a knife during an episode of interpersonal violence. On initial evaluation, she was awake and cooperative but experienced intermittent hemoptysis. Her heart rate was 105 beats per minute, and her blood pressure was 92/48 mmHg. Her respiratory rate was 25 breaths per minute, with a peripheral oxygen saturation (SpO_2_) ranging from 92% to 97% on room air. However, due to the laryngopharyngeal injury, she remained in a semi-upright position and was unable to tolerate the supine posture, raising concerns about impending airway compromise. Physical examination revealed cervical rigidity with marked limitation of neck rotation. Three penetrating soft-tissue wounds were identified on the anterior neck in Zone II, with the largest measuring approximately 15 cm in length (Fig. 1A). During respiration, air bubbles mixed with sputum were observed escaping from the open wound, indicating direct communication with the airway. Further assessment revealed complete transection of the thyroid cartilage, a 2 × 2 cm rupture of the right cricoid cartilage, and laryngotracheal disruption (Fig. 1B and C). No evidence of distal tracheal or esophageal injuries or major vascular hemorrhage was found upon thorough exploration, although multiple cervical muscle transections were present.

An emergency multidisciplinary team (MDT), comprising specialists in trauma surgery, anesthesiology, otolaryngology–head and neck surgery, thoracic surgery, and vascular surgery, was convened promptly. Following MDT discussion, the patient was urgently transferred to the operating room within approximately half an hour for surgical exploration and definitive management of the laryngotracheal injury. Conventional oral or nasal intubation, as well as rapid sequence intubation (RSI), was considered unsafe because of severe anatomical disruption and the associated risk of false passage formation. An awake tracheotomy was also deemed unsuitable owing to anticipated poor patient tolerance related to procedural discomfort. On this basis, awake direct tracheal intubation through the open cervical wound was selected as the airway management strategy. After preoxygenation for three minutes, 2% lidocaine (3 ml) was applied directly to the exposed laryngeal structures via the cervical wound to provide local anesthesia, and a low dose of intravenous midazolam (2 mg) was administered to provide anxiolysis while maintaining spontaneous ventilation. A 7.0 reinforced endotracheal tube was subsequently introduced through the open wound at the level of the transected thyroid cartilage and carefully advanced into the distal trachea. Bilateral symmetric breath sounds were confirmed immediately, and correct intratracheal tube placement was verified using fiberoptic bronchoscopy (Fig. 1D). Further bronchoscopic evaluation and surgical exploration revealed no injuries to the distal trachea or esophagus. During intubation through the wound, the patient’s SpO_2_ level dropped to a minimum of 86% but rapidly returned to 100% after ventilator support was initiated.

After the induction of general anesthesia, the patient was placed on mechanical ventilation. Once airway and hemodynamic stability were achieved, a formal tracheostomy was performed at the level of the second to third tracheal rings. The entire procedure, from intubation to tracheostomy, took approximately 10 min. A size 7 tracheostomy tube was inserted and securely fixed to maintain airway patency, and the initial endotracheal tube was removed (Fig. 1E). Definitive surgical repair included closure of the cricoid cartilage defect, repair of the laryngeal mucosa, and anatomical reconstruction of the thyroid cartilage (Fig. 1F). The thyroid and cricoid cartilages were anatomically reconstructed using 3 − 0 absorbable Vicryl sutures to ensure stable alignment. The laryngeal mucosa was repaired with 4 − 0 Vicryl sutures to promote healing. No grafts or fibrin sealants were used.

**Fig. 2:**
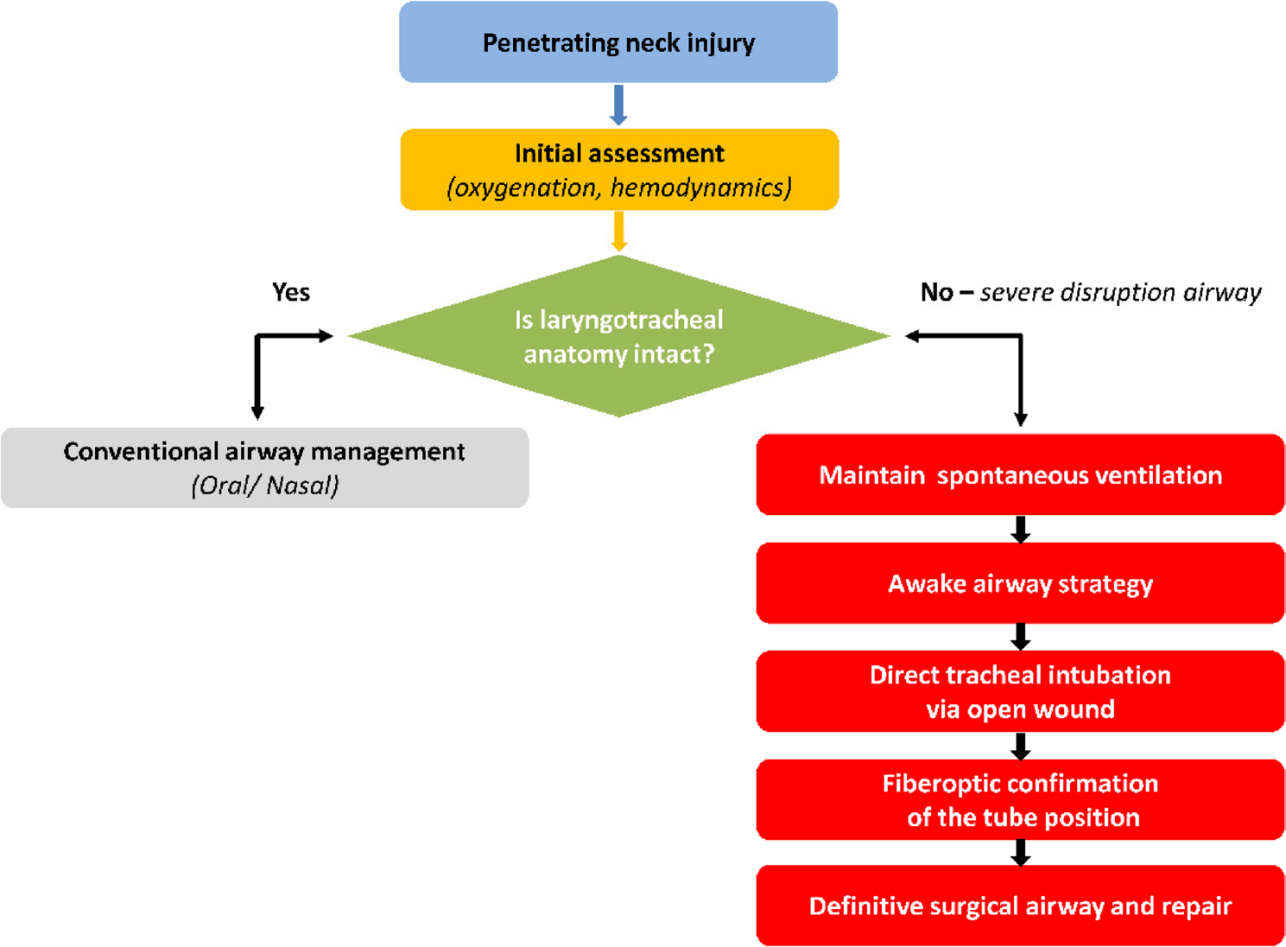
Flowchart of airway management decision-making in severe penetrating neck injury

Postoperatively, the patient was transferred to the intensive care unit, where the clinical course was uneventful. Four weeks later, the tracheostomy was successfully closed by an otolaryngologist following a bronchoscopic examination that confirmed satisfactory airway healing without stenosis. The patient was subsequently followed up in the otolaryngology clinic at three and six months post-surgery. Flexible laryngoscopy revealed smooth mucosa, normal epiglottic elevation, intact bilateral vocal cord mobility, and no visible tracheal stenosis. No signs of hoarseness or other vocal issues were noted throughout the follow-up period. The airway management decision-making process is illustrated in Fig. 2.

## Discussion

Severe penetrating laryngotracheal injuries represent one of the most challenging airway emergencies in trauma care, particularly when profound anatomical disruption occurs in a conscious patient with preserved spontaneous ventilation. In such scenarios, the central clinical dilemma lies not merely in securing the airway but in doing so without precipitating sudden airway loss, false passage formation, or irreversible hypoxic injury [3].

In patients with PNIs, several airway management strategies are theoretically available, including conventional orotracheal or nasotracheal intubation, RSI, emergency cricothyrotomy or tracheostomy, and awake intubation techniques [4, 5]. However, the feasibility and safety of each option depend critically on the integrity of laryngotracheal continuity, the degree of anatomical disruption, and the patient’s physiological tolerance. In the present case, complete transection of the thyroid cartilage, rupture of the cricoid cartilage, and loss of normal airway alignment fundamentally altered the risk-benefit profile of standard airway management approaches.

RSI has been reported as a feasible option in selected PNIs when airway structures and their anatomical relationships remain relatively intact [6–8]. Nevertheless, its application in the setting of extensive laryngotracheal disruption remains highly controversial. Systematic reviews and observational studies have demonstrated that traumatic airway distortion, blood contamination, and soft-tissue disruption are major predictors of first-pass intubation failure [4, 9]. Importantly, failed or repeated intubation attempts are not benign events; they are associated with increased morbidity and may themselves worsen airway injury and edema [10]. Of particular concern, previous reports have documented catastrophic airway failure when an endotracheal tube inserted during RSI penetrated a laryngotracheal defect and created a false passage outside the tracheal lumen, resulting in complete loss of distal airway control [11]. In the presence of disrupted airway continuity, RSI therefore carries not only a high likelihood of technical failure but also a disproportionate risk of irreversible airway catastrophe. For these reasons, RSI was not considered an acceptable option for the present patient.

Preservation of spontaneous ventilation is a cornerstone principle in managing anticipated difficult airways, especially in trauma patients with unstable airway anatomy. Awake airway techniques are generally recommended under such circumstances, as they maintain ventilation while minimizing the risk of sudden airway collapse during induction [12, 13]. However, in this case, conventional awake orotracheal or nasotracheal intubation was also considered unsafe because it significantly increased the risk of tube misdirection, further tissue disruption, and false passage formation, even when advanced visualization techniques were available. Thus, although awake intubation is often advocated in difficult airway scenarios, its traditional routes were not suitable in this specific anatomical context.

In addition, fiberoptic-guided intubation was limited by the absence of a suction channel and the active expulsion of secretions and blood, which quickly obscured the visual field. Under these conditions, direct tracheal intubation through the thyroid cartilage fracture was performed while simultaneous suction was applied to maintain airway patency. Fiberoptic bronchoscopy was subsequently used to confirm correct tube placement once an adequate visual field had been established. This approach allowed for rapid and controlled airway access in a highly challenging anatomical setting.

Although emergency tracheostomy or cricothyrotomy is frequently advocated as a definitive airway in patients with traumatic airway compromise [14, 15], these procedures can be technically challenging and poorly tolerated in awake patients [16]. In the present case, severe respiratory discomfort prevented the patient from lying flat, and extensive cervical distortion further increased procedural uncertainty. Collectively, these factors rendered an awake tracheostomy both technically hazardous and physiologically impractical during the initial phase of airway management.

Given these considerations, an individualized airway strategy prioritizing preservation of spontaneous ventilation and direct control of the distal airway was required. Awake tracheal intubation through the open cervical wound offered a unique solution in this context. The existing laryngotracheal disruption provided immediate access to the distal tracheal lumen, allowing the endotracheal tube to be introduced under direct visualization and minimizing the risk of false passage formation [6]. After intubation, the tube’s position can be confirmed via bronchoscopy to ensure accurate placement. This approach enabled rapid and controlled airway stabilization while avoiding the risks associated with both RSI and conventional awake orotracheal or nasotracheal intubation.

In the event of failed intubation through the open cervical wound, alternative airway strategies were anticipated. Emergency tracheostomy under local anesthesia was considered the definitive backup approach. To facilitate rapid airway access, the distal segment of the fractured thyroid cartilage was secured with silk sutures to serve as an anatomical landmark and to prevent retraction of the distal trachea. Additionally, high-flow nasal oxygen was prepared in advance to reduce the risk of hypoxemia during airway management.

Adequate airway anesthesia and cautious sedation were essential to the success of this strategy. Topical lidocaine applied directly to the exposed tracheal mucosa provided effective local anesthesia, consistent with established recommendations for awake tracheal intubation. Minimal sedation with low-dose midazolam was deliberately selected to provide anxiolysis while preserving spontaneous ventilation and protective airway reflexes. Other agents, including propofol, opioids, and dexmedetomidine, were considered suboptimal due to their potential to cause respiratory depression, loss of airway reflexes, or hemodynamic instability—events that could be catastrophic in a patient with severely disrupted airway anatomy [2].

Although awake tracheal intubation through the open cervical wound is not included in standard difficult airway algorithms, it has been described as a life-saving bridging technique in severe PNIs. When an open laryngotracheal disruption is present and distal airway access can be achieved under direct visualization, this approach may offer a safer alternative to blind or forceful manipulation of disrupted structures. Importantly, this strategy should be regarded as a temporary measure, facilitating a controlled transition to a definitive airway—most commonly via a formal tracheostomy performed inferior to the site of injury [17–21].

The present case further underscores the importance of MDT in complex airway emergencies. The decision to proceed with awake direct tracheal intubation through the cervical wound was reached by rapid consensus among anesthesiology, trauma surgery, and otolaryngology specialists, rather than by individual operator preference. Such coordinated decision-making is increasingly recognized as a critical factor in successful airway management and improved outcomes in high-risk trauma patients [22].

While successful decannulation at four weeks indicates a favorable short-term outcome, ongoing dynamic airway surveillance remains essential in cases of laryngotracheal disruption and reconstruction. Post-intubation granulation tissue formation and scar-related stenosis of the laryngotracheal segment remain significant potential complications [23]. Scar-related stenosis at the laryngotracheal junction may develop progressively and, in severe cases, require complex secondary reconstruction. Previous reports have highlighted the challenges associated with delayed laryngotracheal reconstruction following trauma [24, 25]. Therefore, long-term follow-up with sequential endoscopic evaluations is recommended to facilitate early detection and timely management of late airway complications.

Despite the favorable outcome, several limitations must be acknowledged. The patient did not undergo a preoperative computed tomography (CT) scan and instead proceeded directly to intraoperative exploration due to the urgent need for airway stabilization. However, the patient did receive an additional head and neck CT examination after surgery to ensure no misdiagnosis. As a single case, the observations cannot be generalized to all penetrating laryngotracheal injuries. Moreover, this technique requires favorable anatomical conditions, advanced technical expertise, and immediate MDT support, which may not be universally available. Therefore, awake tracheal intubation through the open cervical wound should not be considered a routine airway management strategy, but rather a carefully selected option in highly specific clinical circumstances.

In conclusion, this case demonstrates that in severe PNIs, standard airway algorithms may be insufficient or unsafe. An individualized, anatomy-driven approach that prioritizes the preservation of spontaneous ventilation and direct control of the distal airway is critical for successful management. Awake tracheal intubation through the open cervical wound can serve as an effective bridging technique to definitive airway reconstruction in carefully selected patients. This case highlights not only the feasibility of an unconventional airway approach but also the importance of structured long-term follow-up to prevent delayed airway complications.

## Supplementary Information

Supplementary Material 1.

## Data Availability

No datasets were generated or analysed during the current study.

## Abbreviations

CT: Computed tomography
ED: Emergency department
ICU: Intensive care unit
MDT: Multidisciplinary team
PNIs: Penetrating neck injuries
RSI: Rapid sequence intubation

## References

[1] Forner D, Noel CW, Guttman MP, Haas B, Enepekides D, Rigby MH, et al. Blunt versus penetrating neck trauma: A retrospective cohort study. Laryngoscope. 2021;131:E1109–16. 10.1002/lary.29088. 32894596 10.1002/lary.29088

[2] Singerman KW, Kavookjian HL, Kraft SM. Evaluation and management of blunt and penetrating laryngopharyngeal injuries. Facial Plast Surg Clin North Am. 2025;33:421–35. 10.1016/j.fsc.2025.03.013. 40581459 10.1016/j.fsc.2025.03.013

[3] Loss L, Henry R, White A, Matsushima K, Barrett C, Lammers D, et al. Penetrating neck trauma: a comprehensive review. Trauma Surg Acute Care Open. 2025;10:e001619. 10.1136/tsaco-2024-001619. 40166772 10.1136/tsaco-2024-001619 PMC11956299

[4] Mercer SJ, Jones CP, Bridge M, Clitheroe E, Morton B, Groom P. Systematic review of the anaesthetic management of non-iatrogenic acute adult airway trauma. Br J Anaesth. 2016;117(Suppl 1):i49–59. 10.1093/bja/aew193. 27566791 10.1093/bja/aew193

[5] Piaseczny M, La J, Chaplin T, Evans C. Protect that neck! Management of blunt and penetrating neck trauma. Emerg Med Clin North Am. 2023;41:35–49. 10.1016/j.emc.2022.09.005. 36424043 10.1016/j.emc.2022.09.005

[6] Ahmad I, El-Boghdadly K, Bhagrath R, Hodzovic I, McNarry AF, Mir F, et al. Difficult Airway Society guidelines for awake tracheal intubation (ATI) in adults. Anaesthesia. 2020;75:509–28. 10.1111/anae.14904. 31729018 10.1111/anae.14904 PMC7078877

[7] Jain U, McCunn M, Smith CE, Pittet J-F. Management of the traumatized airway. Anesthesiology. 2016;124:199–206. 10.1097/ALN.0000000000000903. 26517857 10.1097/ALN.0000000000000903

[8] Ahmad I, El-Boghdadly K, Iliff H, Dua G, Higgs A, Huntington M, et al. Difficult airway society 2025 guidelines for management of unanticipated difficult tracheal intubation in adults. Br J Anaesth. 2025. 10.1016/j.bja.2025.10.006. :S0007-912(25)693-2. 41203471 10.1016/j.bja.2025.10.006

[9] Simpson C, Tucker H, Griggs J, Gavrilovski M, Lyon R, Hudson A, et al. Pre-hospital management of penetrating neck injuries: Derivation of an algorithm through a national modified delphi. Scand J Trauma Resusc Emerg Med. 2024;32:123. 10.1186/s13049-024-01291-1. 39623494 10.1186/s13049-024-01291-1 PMC11613838

[10] Hayes-Bradley C, McCreery M, Delorenzo A, Bendall J, Lewis A, Bowles K-A. Predictive and protective factors for failing first pass intubation in prehospital rapid sequence intubation: An aetiology and risk systematic review with meta-analysis. Br J Anaesth. 2024;132:918–35. 10.1016/j.bja.2024.02.004. 38508943 10.1016/j.bja.2024.02.004

[11] Tian J, Tao X, Quan X, Zhang S. What we have learned from a patient with partial tracheal rupture caused by penetrating neck injuries: A case report. BMC Anesthesiol. 2022;22:333. 10.1186/s12871-022-01886-0. 36316640 10.1186/s12871-022-01886-0 PMC9623958

[12] Apfelbaum JL, Hagberg CA, Connis RT, Abdelmalak BB, Agarkar M, Dutton RP, et al. 2022 American society of anesthesiologists practice guidelines for management of the difficult airway. Anesthesiology. 2022;136:31–81. 10.1097/ALN.0000000000004002. 34762729 10.1097/ALN.0000000000004002

[13] Kovacs G, Sowers N. Airway management in trauma. Emerg Med Clin North Am. 2018;36:61–84. 10.1016/j.emc.2017.08.006. 29132582 10.1016/j.emc.2017.08.006

[14] Pincet L, Lecca G, Chrysogelou I, Sandu K. External laryngotracheal trauma: A case series and an algorithmic management strategy. Eur Arch Otorhinolaryngol. 2024;281:1895–904. 10.1007/s00405-024-08456-9. 10.1007/s00405-024-08456-9 PMC10943164 38261015

[15] Haran C, Kong V, Cheung C, Rajaretnam N, Bruce J, Laing G, et al. Managing the acutely threatened airway following head and neck trauma - requiem for cricothyroidotomy? Surg Pract Sci. 2022;11:100124. 10.1016/j.sipas.2022.100124. 39845158 10.1016/j.sipas.2022.100124 PMC11749900

[16] Shilston J, Evans DL, Simons A, Evans DA. Initial management of blunt and penetrating neck trauma. BJA Educ. 2021;21:329–35. 10.1016/j.bjae.2021.04.002. 34447579 10.1016/j.bjae.2021.04.002 PMC8377225

[17] Soundararajan AS, Krishna KP. Lifesaving surgical approaches for severe penetrating knife injury to the neck. J Cardiothorac Surg. 2025;20:97. 10.1186/s13019-024-03233-5. 39865304 10.1186/s13019-024-03233-5 PMC11771049

[18] Asimakopoulos AD, Sandu K. High-impact laryngotracheal trauma: a combined narrative and systematic review evaluating gaps in the current laryngeal injury reporting system. ORL J Otorhinolaryngol Relat Spec. 2025:1-12. 10.1159/000549780. 10.1159/000549780 PMC12818889 41343417

[19] NaqviSayyed EH, Sadik A, Beg MH, Azam H, Nadeem R, Eram A. Successful management of suicidal cut throat injury with internal jugular, tracheal and esophageal transection: A case report. Trauma Case Rep. 2017;13:30–4. 10.1016/j.tcr.2017.11.005. 29644295 10.1016/j.tcr.2017.11.005 PMC5887116

[20] Dai G, Yan X. Emergency management of cut throat injury: A report of 2 cases. Am J Case Rep. 2025;26:e946414. 10.12659/AJCR.946414. 39962797 10.12659/AJCR.946414 PMC11843778

[21] Aljohani K, Alsaud A, Aldarsouni FG, Alruwaite H, Alsubaie NM. Penetrating neck injury: Double jeopardy of a complex aerodigestive dilemma. Cureus 15:e39533. 10.7759/cureus.39533 10.7759/cureus.39533 PMC10290894 37366441

[22] Lin LC, April MD, Douin DJ, Winkle JM, Jenson WR, Rizzo JA, et al. Airway management in trauma patients: A seven-year review of emergency department intubations. Am J Emerg Med. 2026;99:306–12. 10.1016/j.ajem.2025.10.023. 41135359 10.1016/j.ajem.2025.10.023 PMC13399005

[23] Topolnitskiy E, Chekalkin T, Marchenko E, Volinsky A. Treatment of post-resuscitation cicatricial tracheal stenosis after suffering severe COVID-19 associated pneumonia: a report of 11 cases. Respir Med Case Rep. 2022;40:101768. 10.1016/j.rmcr.2022.101768. 36312301 10.1016/j.rmcr.2022.101768 PMC9597538

[24] Syal R, Tyagi I, Goyal A. Traumatic laryngotracheal stenosis–an alternative surgical technique. Int J Pediatr Otorhinolaryngol. 2006;70:353–7. 10.1016/j.ijporl.2005.06.023. 16102847 10.1016/j.ijporl.2005.06.023

[25] Topolnitskiy EB, Shefer NA, Podgornov VF. [a non-standard approach in the treatment of post-traumatic multifocal cicatricial tracheal stenosis with atresia of subglottic larynx, involvement of vocal cords and 33-year cannulation]. Vestn Otorinolaringol. 2022;87:113–7. 10.17116/otorino202287041113. 36107191 10.17116/otorino202287041113

